# Multiparental Mapping of Plant Height and Flowering Time QTL in Partially Isogenic Sorghum Families

**DOI:** 10.1534/g3.114.013318

**Published:** 2014-09-01

**Authors:** R. H. Higgins, C. S. Thurber, I. Assaranurak, P. J. Brown

**Affiliations:** * Department of Crop Sciences, University of Illinois, Urbana, Illinois 61801; † Energy Biosciences Institute, University of Illinois, Urbana, Illinois 61801

**Keywords:** genetic heterogeneity, allelic series, photoperiod, linkage, GWAS, Multiparent Advanced Generation Inter-Cross (MAGIC), multiparental populations, MPP

## Abstract

Sorghum varieties suitable for grain production at temperate latitudes show dwarfism and photoperiod insensitivity, both of which are controlled by a small number of loci with large effects. We studied the genetic control of plant height and flowering time in five sorghum families (A–E), each derived from a cross between a tropical line and a partially isogenic line carrying introgressions derived from a common, temperate-adapted donor. A total of 724 F_2:3_ lines were phenotyped in temperate and tropical environments for plant height and flowering time and scored at 9139 SNPs using genotyping-by-sequencing. Biparental mapping was compared with multiparental mapping in different subsets of families (AB, ABC, ABCD, and ABCDE) using both a GWAS approach, which fit each QTL as a single effect across all families, and using a joint linkage approach, which fit QTL effects as nested within families. GWAS using all families (ABCDE) performed best at the cloned *Dw3* locus, whereas joint linkage using all families performed best at the cloned *Ma1* locus. Both multiparental approaches yielded apparently synthetic associations due to genetic heterogeneity and were highly dependent on the subset of families used. Comparison of all mapping approaches suggests that a GA2-oxidase underlies *Dw1*, and that a mir172a gene underlies a *Dw1*-linked flowering time QTL.

Sorghum (*Sorghum bicolor*) is a tropical C_4_ grass with great resilience to abiotic and biotic stress that can be used to produce grain, sugar, and biomass. Most sorghum varieties are tall and photoperiod-sensitive, requiring day-lengths of less than 12 hr to initiate flowering, but most sorghum breeding has focused on short, photoperiod-insensitive varieties suitable for grain production at temperate latitudes. Genetic improvement of sorghum as a temperate bioenergy crop and a tropical grain crop is constrained by limited genetic exchange between elite temperate and diverse exotic gene pools. Understanding the genetic control of dwarfing and photoperiod insensitivity in sorghum will help it achieve its full potential as a source of both food security in tropical developing nations and clean, renewable bioenergy in the industrialized world.

Dwarfing and photoperiod-insensitive early maturity in sorghum are each controlled by at least four major loci (*Dw1-4* and *Ma1-4*) (Quinby 1974). *Dw3* encodes an auxin efflux carrier, PGP19 (Multani *et al.* 2003), and *Ma1* encodes a pseudo-response regulator protein, PRR37 (Murphy *et al.* 2011). Additional maturity loci with large effects have been reported (*Ma5-6*) (Rooney and Aydin 1999). Genetic diversity and phenotypic diversity in dwarf, photoperiod-insensitive sorghum have been greatly increased by the Sorghum Conversion Program, which used backcrossing with selection to introgress elite genomic segments into diverse exotic accessions (Figure 1A) (Klein *et al.* 2008). Molecular evidence suggests that introgressions in the resulting sorghum converted (SC) lines are concentrated in three genomic regions containing *Dw3*, *Ma1*, and the uncloned *Dw1* locus (Thurber *et al.* 2013). However, each of these regions appears to contain multiple QTL for plant height and maturity and/or multiple functional alleles at each locus (Murphy *et al.* 2011; Barrero Farfan *et al.* 2012; Thurber *et al.* 2013). Lower-frequency introgressions outside these regions might contain additional QTL for plant height and flowering time, or QTL for unintentionally selected traits such as seed dormancy and fertility restoration (Thurber *et al.* 2013).

**Figure 1:**
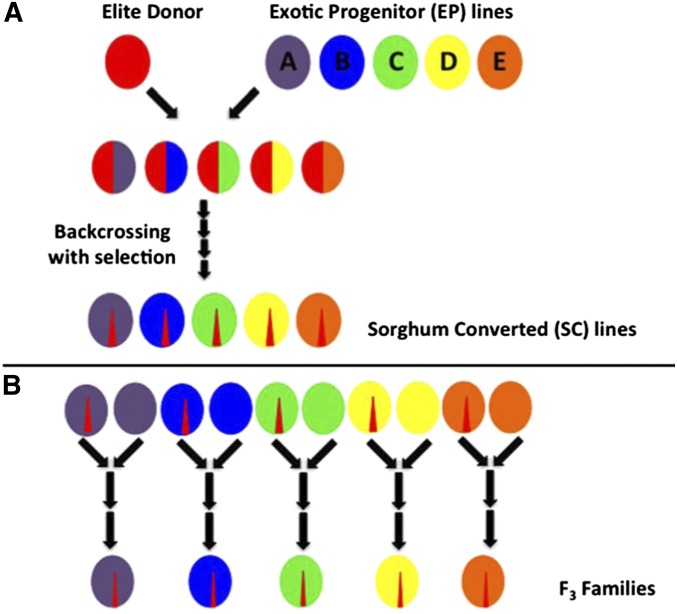
Creation of partially isogenic F_3_ families. (A) A short, early-flowering elite donor line was crossed to multiple tall, late, exotic progenitor (EP) lines, and multiple backcrosses were performed to the EP lines while selecting for early flowering and short stature. The resulting sorghum conversion (SC) lines contain small introgressions of the elite donor in the background of the EP. (B) Five SC lines were crossed to their corresponding EP lines to create a total of 724 F_3_ lines from five families.

Population structure in cultivated sorghum corresponds closely to the morphologically defined races of cultivated sorghum: *durra*, *caudatum*, *guinea*, *kafir*, and *bicolor* (Brown *et al.* 2011a). Different races harbor different loss-of-function alleles at the *Sh1* locus responsible for loss of seed shattering in cultivated sorghum (Lin *et al.* 2012). More than any other trait, loss of seed shattering distinguishes domesticated grasses from wild grasses (Meyer and Purugganan 2013), suggesting the occurrence of multiple domestication events in sorghum with different races arising from genetically divergent populations of wild sorghum.

Almost every quantitative trait locus identified in sorghum displays allelic genetic heterogeneity, with multiple mutations in the same gene conferring similar phenotypes. In addition to the three characterized knockout alleles at *Sh1*, two alleles at *Dw3* confer dwarfism (Multani *et al.* 2003; Barrero Farfan *et al.* 2012), two alleles at *Tan1* confer loss of tannins in the pericarp (Wu *et al.* 2012), and a half-dozen distinct alleles at *Ma1* confer photoperiod insensitivity (Murphy *et al.* 2011; R. Klein, personal communication). Such genetic heterogeneity complicates marker–trait association analyses and can generate synthetic associations (Brachi *et al.* 2011; Lin *et al.* 2012).

Multiparental mapping strategies occupy a middle ground between the limited inference space of biparental mapping and the complex population structure inherent to association mapping. In maize, several recent studies have made use of the nested association mapping (NAM) population of maize RILs, which was created by crossing 26 diverse inbreds to a single female parent (McMullen *et al.* 2009). Several multiparental models have been developed, including a “joint linkage” approach in which a modest number of markers are genotyped and QTL are fit as terms nested within families (Buckler *et al.* 2009) and a “GWAS” approach in which a vast number of markers are genotyped in the parental lines, projected onto the progeny, and used to fit QTL as non-nested terms (Tian *et al.* 2011). Both approaches have been applied to the genetic dissection of leaf (Tian *et al.* 2011), inflorescence (Brown *et al.* 2011b), disease resistance (Poland *et al.* 2011; Kump *et al.* 2011), kernel composition (Cook *et al.* 2012), stalk strength (Peiffer *et al.* 2013), and plant height (Peiffer *et al.* 2014) traits in the maize NAM population. The two models differ in their approach to the problem of distinguishing allelic series from linked genes: the nested effects fit by the joint linkage approach are likely to fuse closely linked QTL, whereas the non-nested effects fit by the GWAS approach are likely to split allelic series. The first multiparental mapping study in sorghum applied two similar mapping approaches to identify flowering time QTL in hybrids in a large backcross (BC) NAM population of 24 families crossed to a common tester (Mace *et al.* 2013). All these multiparental studies share a reference design, in which all lines evaluated share at least half of their genome with a common parent.

In this study, we applied joint linkage and GWAS approaches to map plant height and flowering time QTL in a population of five sorghum F_3_ families (A–E). Each family was created by crossing a dwarf, photoperiod-insensitive SC line to its corresponding exotic progenitor, which is expected to be isogenic for the entire genome except for the introgressed QTL regions (Figure 1B) (Thurber *et al.* 2013). Genetically divergent SC lines were chosen to represent different racial groups in sorghum. Therefore, our population is expected to show reduced within-family variance and increased between-family variance relative to a reference design. F_3_ families were phenotyped for plant height and flowering time in a temperate environment, where segregation for photoperiod sensitivity dramatically affected both traits, and in a tropical environment, where the effects of photoperiod sensitivity were minimal. Biparental mapping was performed in each family separately, and the two multiparental mapping approaches were performed in five subsets of families with increasing levels of genetic divergence: AB, ABC, ABCD, and ABCDE. Inclusion of more families increases sample size and is expected to increase power and resolution, but these advantages may be compromised by increasing genetic heterogeneity. We then assessed the success of these different approaches using the cloned *Dw3* and *Ma1* loci, which have large effects on plant height and flowering time in sorghum.

## Materials and Methods

### Plant materials and growing conditions

Biparental families A–E were created by crossing five SC lines to their five corresponding exotic progenitor (EP) lines (Figure 1). SC lines are short, early-flowering lines used in grain sorghum breeding, and EP lines are tall, late-flowering exotic landrace accessions. Each SC line was created from an EP line by crossing to a short, early-flowering elite donor (BT×406) and then using the EP line as the recurrent parent over at least four cycles of backcrossing with selection for short stature and early flowering. Because families A–E are derived from paired SC×EP crosses, they are expected to be partially isogenic (Figure 1).

Seed for the five SC lines was obtained from the USDA-ARS Cropping Systems Research Laboratory (Lubbock, TX) and seed for the five corresponding EP lines was obtained from the National Plant Germplasm System. Family A was derived from SC673, a *kafir* from Zimbabwe. Family B was derived from SC757, a *kafir* from Botswana. Family C was derived from SC1203, a *caudatum* from Brazil. Family D was derived from SC1038, a *durra* from Ethiopia. Family E was derived from SC991, a *bicolor* from Uganda. Initial crosses between SC and EP lines and self-pollination of one to two F_1_ individuals per cross were both performed in the greenhouse after photoperiod induction for 10 wk of 12-hr days in a growth chamber. F_2_ individuals from each family were grown in 12 randomized 6-m rows, selfed, and phenotyped in the winter of 2011–2012 near San Jose del Valle, Nayarit, Mexico, a tropical environment (20.7**°**N). One hundred ninety-two random F_3_ entries from each family were grown and phenotyped in the summer of 2012 in Urbana, Illinois, a temperate environment (40.1**°**N) in 6-m rows. Each family was split into four blocks of 48 lines, and their position was randomized across the field. Both parental lines were included in each block, for a total of 200 rows per family.

### Phenotyping

Plant height was measured in centimeters to the apex, and flowering time was measured in days from planting to the initiation of anthesis. In Mexico, plant height (HT-MX) and flowering time (FL-MX) phenotypes were collected from individual F_2_ plants. In Urbana, Illinois, plant height (HT-IL) and flowering time (FL-IL) phenotypes represent the mean of each F_3_ row. Three of five families (C, D, and E) segregated for a dominant photoperiod sensitivity allele that caused long delays in flowering in Urbana. To obtain meaningful estimates of mean flowering time from F_3_ rows segregating for a large-effect photoperiod sensitivity locus, we scored the dates at which each row reached 25% anthesis and 75% anthesis and scored FL-IL as the mean of these two dates. The raw and calculated values for temperate days to anthesis (FL-IL) are shown in Supporting Information, Table S1. Individuals that had not reached anthesis by the killing frost were assigned a flowering date of 138 days, and plant height to the apex was recorded even if the apex was a leaf whorl instead of a panicle. This practical approximation only slightly underestimates the effects of photoperiod sensitivity on plant height in our study. One hundred fifteen rows contained at least 25% of individuals in this category, and nine rows contained 100% of individuals in this category. Phenotypic models were fit using the lmer function in R (R Core Team 2013). For HT-MX and FL-MX, family, row, and genotype were fit as random effects. For HT-IL and FL-IL, family, block, and genotype were fit as random effects. Genotype explained no variance in the FL-MX phenotype, so it was excluded from further analysis (Table S3). The lack of replication and the single tropical and temperate environments used in this study are justified on the grounds that the QTL effects of interest are very large, with additive effects of approximately 5 to 20 d for flowering time and 20 cm to 75 cm for plant height, and the genetic architecture of these traits in partially isogenic families expected to be relatively simple.

### Genotyping and SNP calling

Genomic DNA was extracted from five etiolated seedlings from each F_3_ line using a modified CTAB protocol (Thurber *et al.* 2013), quantified using PicoGreen (Invitrogen, NY), and genotyped using genotyping-by-sequencing (GBS). To create GBS libraries, DNAs (∼250 ng) were double-digested with either *PstI*-HF and *BfaI* or *PstI*-HF and *HinP1I* and ligated to one of 384 unique DNA barcodes. The resulting samples were then pooled for amplification and size selection (Thurber *et al.* 2013). Each 384-sample library was submitted to the W. M. Keck Center at the University of Illinois for single-end 100-bp sequencing on the Illumina HiSeq2000, where an additional qPCR assay was performed on each library to adjust concentrations before sequencing.

Four lanes of 100-bp, single-end Illumina reads were obtained. Each lane contained 384 barcodes consisting of 192 genomic DNAs cut with two pairs of restriction enzymes (*PstI-BfaI* and *PstI-HinP1I*), for a total of 768 DNA samples across four lanes. The two parents of each population were replicated twice, for a total of 20 parental samples. The remaining 748 samples consisted of randomly selected F_3_ lines from each family. A total of 724 lines were included in the QTL analysis, with the difference being due to lines with a SNP call rate of less than 10%. These 24 cases (3.2%) were most likely due to poor DNA quality and/or failure of the restriction-ligation reaction. These 724 genotypes are distributed uniformly across the flowering time distribution within each family (Figure S2).

The TASSEL GBS pipeline (Glaubitz *et al.* 2014) was used to process Illumina fastq files and call SNPs using version 2.1 of the sorghum bicolor reference genome (Paterson *et al.* 2009) available at Phytozome (www.phytozome.net). Full-sib family haplotype imputation (FSFHap) implemented in TASSEL 5 (K. Swarts, E. S. Buckler, P. J. Bradbury, unpublished results) was used to identify parental haplotypes and impute heterozygous genotypes and other missing data.

### Genetic segregation within and between families

Families A–E were chosen to represent the different sorghum races: durra, caudatum, kafir, guinea, and bicolor. No guinea families were successfully generated, so we instead included two kafir families that share common ancestry with guineas (Thurber *et al.* 2013). The parents of family E (SC0991×EP0991) were subsequently found to contain a high proportion (25%) of unexpected genotypes (Thurber *et al.* 2013), indicating that they are probably not a nearly isogenic pair. The parents of families A, B, C, and D contained 3%, 8%, 1%, and 14% unexpected genotypes, respectively (Table 1). To provide an independent assessment of whether each of the five families was derived from a cross between the two intended nearly isogenic parents, two tests were performed. First, genotype data from the F_2:3_ lines were used to reconstruct the maternal and paternal haplotypes for each family. These haplotypes could differ from those of the intended parents due to pollen or seed contamination. For each of five families, we calculated the percent identity-by-state of the reconstructed maternal and paternal haplotypes to a set of 1160 of previously genotyped sorghum taxa that included all 10 parents of the families A–E (Thurber *et al.* 2013) and recorded the closest match. Second, hierarchical clustering was performed on the 1160 sorghum taxa using the hclust function and the “complete” method in R to assess whether the two parents of each family were more similar to each other than to any other inbred line in the dataset.

**Table 1:** Genetic analysis of sorghum families A–E

Family	Race	% of Genotypes Matching*^a^*			F_3_s Match Parents	Parents Related	% of Genome Segregating*^b^*
		Elite Donor	Exotic Progenitor	Neither			
A	Kafir	5	92	3	Y	Y	1.3
B	Kafir	10	82	8	Y	Y	5.5
C	Caudatum	13	86	1	Y	Y	15.7
D	Durra	22	64	14	Y	Y	61.3
E	Bicolor	30	45	25	Y	N	80.4

a From Thurber *et al.* (2013).

b Excludes regions >10 Mb with no polymorphic markers.

### Marker–trait association

Initial SNP calling yielded a set of 12,256 SNPs segregating in at least one family. Trimming SNPs with >5% missing data and trimming adjacent, nearby (<64 bp) SNPs with identical genotypes produced a set of 9139 SNPs for further analysis (Table S1). Most of the 9139 polymorphic SNPs segregated in a single population, and no SNPs segregated in all five populations. For linkage mapping in one or more families, a new SNP dataset was created that tracked the parent-of-origin within each family but did not track identity-by-descent between families (Table 2). To create this dataset, SC genotypes were set to 0, EP genotypes were set to 2, monomorphic SNPs within each family were set as missing, and missing data were imputed as the mean of the nearest flanking markers weighted by physical distance. For example, a monomorphic SNP 0.75 kb from an SC genotype and 0.25 kb from an EP genotype would receive a value of 1.5. Nearby SNPs are always in high linkage disequilibrium (LD) with each other in this dataset because it reflects only the meioses that occurred during the creation of each F_2:3_ family. Chromosomes and chromosomal regions larger than 10 Mb with no polymorphic markers in an individual family were assumed not to be segregating and were left as missing data. Multiparental mapping using the GWAS approach used the unmodified SNP dataset, in which some nearby markers are in very low LD with each other due to ancestral recombination events between parental lines and there is virtually no missing data.

**Table 2:** Comparison of GWAS and linkage genotypic datasets

Dataset	Family	Line	SNP1	SNP2	SNP3	SNP4	SNP5
GWAS	A	1	2	**0**	2	**0**	1
	A	2	1	**0**	1	**0**	1
	A	3	0	**0**	1	**0**	1
	B	1	1	**0**	**2**	2	2
	B	2	1	**0**	**2**	1	2
	B	3	0	**0**	**2**	0	0
Linkage	A	1	2	**2**	2	**1.5**	1
	A	2	1	**1**	1	**1**	1
	A	3	0	**0.5**	1	**1**	1
	B	1	1	**1.34**	**1.67**	2	2
	B	2	1	**1**	**1**	1	2
	B	3	0	**0**	**0**	0	0

Five hypothetical, equidistant SNPs from two families are presented. SNPs monomorphic within families are shown in bold and are imputed as the mean of the two flanking markers in the joint linkage dataset

All marker–trait association was performed by Haley-Knott regression using the lm function in R. Biparental mapping in individual families was performed with the linkage dataset using forward regression with significance determined by 1000 permutations and a genome-wide α level of 0.01. Multiparental mapping was performed using forward regression with family as a covariate and by fitting SNP effects as nested within family using the linkage genotypic dataset or by fitting SNPs as non-nested effects using the GWAS genotypic dataset. Significance thresholds were determined by 1000 permutations of phenotypes within each family separately using a genome-wide α level of 0.01.

## Results and Discussion

### Genetic and phenotypic variation within and between families

**Figure 2:**
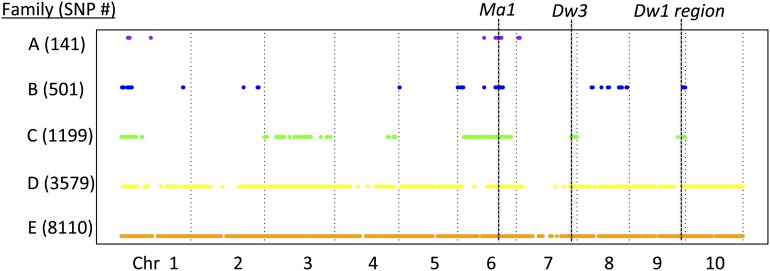
Genetic segregation in F_3_ families. Polymorphic SNPs segregating in each family are indicated by colored circles across the sorghum genome. Families A, B, and C are derived from partially isogenic parents, family D is derived from related parents, and family E is derived from unrelated parents.

**Figure 3:**
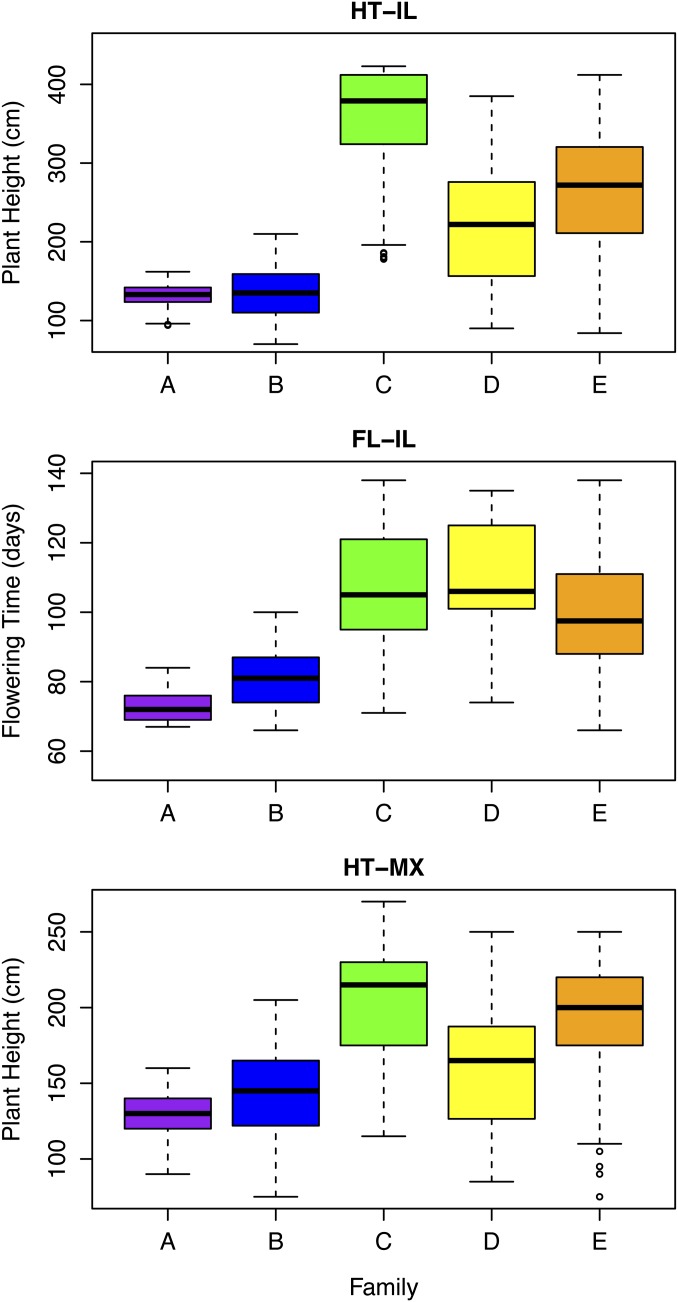
Phenotypic segregation in F_3_ families. Plant height (HT) and flowering time (FL) were measured in a temperate environment in Illinois (IL), and plant height was measured in a tropical environment in Mexico (MX). Box edges mark the first and third quartiles, whiskers extend up to 1.5-times the interquartile range, and outliers are shown as circles.

Three of five biparental families (A, B, and C) are nearly isogenic, segregating for an estimated 1.3%–15.7% of the genome (Figure 2). This is within the range of segregation expected after four generations of backcrossing with selection for oligogenic traits. The other two biparental families (D and E) segregate for SNPs on every chromosome arm, suggesting that the parents were not closely related. Reconstructed maternal and paternal haplotypes from each family closely match their presumed maternal and paternal parents on every chromosome, indicating that pollen or seed contamination did not occur during the creation of the families. Hierarchical clustering reveals that the parents of families A–D are more similar to each other than to any other taxon in a dataset of 1160 sorghum accessions, whereas the parents of family E are not closely related. Together, these results indicate that family D is derived from a cross between the intended partially isogenic parents, but family E is not. The large number of SNPs segregating in family D compared with families A, B, and C could reflect greater residual heterozygosity in the original exotic accession, followed by genetic drift in the lineages leading to the parents of family D. Family E can be viewed as an essentially random cross between a tall photoperiod-sensitive sorghum line and a dwarf photoperiod-sensitive sorghum line. Three of five biparental families (C, D, and E) segregated for a strong photoperiod sensitivity response that resulted in dramatically greater variance in flowering time and plant height in the temperate environment (Figure 3). The other two families (A and B) are both derived from the *kafir* race of sorghum, which predominates in temperate latitudes of southern Africa and possesses a distinct loss-of-function allele at the *Ma1* locus (*Sbprr37-2*) (Murphy *et al.* 2011). Altogether, our results suggest that families A–E represent a gradient of increasing genetic and phenotypic segregation: families A and B are nearly isogenic and photoperiod-insensitive; family C is nearly isogenic but photoperiod-sensitive; family D is photoperiod-sensitive with related parents; and family E is photoperiod-sensitive with unrelated parents.

### Comparison of biparental and multiparental models

The five biparental and eight multiparental models for each of the three traits are summarized in Table 3. Segregation of large-effect QTL in partially isogenic families produced highly predictive QTL models. Biparental models for plant height and flowering time in the temperate environment (HT-IL and FL-IL) contained 1–3 QTL that explained 65%–84% of the phenotypic variance. Models for plant height in the tropical environment (HT-MX) explained far less variance. This is likely due to more precise phenotyping of F_3_ rows in Illinois compared with F_2_ individuals in Mexico, to greater additive genetic variance in the F_3_ compared with the F_2_ generation, and to increased phenotypic variance in Illinois compared with Mexico due to segregation for photoperiod sensitivity. For example, FL-IL QTL linked to the major photoperiod sensitivity locus *Ma1* usually explained more than 70% of the phenotypic variance in an individual family. However, models for temperate and tropical plant height often shared markers, and the difference in variance explained by these models grew smaller as more families were included in the analysis and more between-family variance was explained by the family covariate. Joint linkage multiparental models generally included fewer terms but explained the same amount of variance as GWAS models.

**Table 3:** Variance explained (r^2^_adj_) and number of QTL for each trait and model

Families	Model	Trait					
		HT-IL		FL-IL		HT-MX	
		r^2^	# QTL	r^2^	# QTL	r^2^	# QTL
Multiparental							
ABCDE	GWAS	0.90	5	0.89	5	0.89	6
ABCDE	Linkage	0.90	4	0.90	3	0.90	3
ABCD	GWAS	0.92	5	0.91	5	0.90	6
ABCD	Linkage	0.92	3	0.89	3	0.89	3
ABC	GWAS	0.95	4	0.91	3	0.91	4
ABC	Linkage	0.94	3	0.89	3	0.91	3
AB	GWAS	0.79	3	0.77	3	0.56	2
AB	Linkage	0.79	3	0.77	2	0.57	2
Biparental							
A	Linkage	0.84	2	0.68	1	0.16	1
B	Linkage	0.78	3	0.69	2	0.52	2
C	Linkage	0.50	2	0.75	2	0.19	2
D	Linkage	0.69	2	0.76	1	0.50	2
E	Linkage	0.65	3	0.70	1	0.26	1

### Incidence of major QTL for plant height and flowering time

All five families segregate for a flowering time QTL in the *Ma1* region on chromosome 6, and four families segregate for a plant height QTL in the linked *Dw2* region. In addition, three families segregate for a plant height QTL in the *Dw1* region on chromosome 9, two families segregate for a flowering time QTL in the linked *SbFL9.1* region, and two families segregate for plant height QTL in the *Dw3* region on chromosome 7 (Table 4). For every major QTL except *Ma1*, there are families that segregate for introgressions, but not QTL, in that region (*Dw2* in family C, *Dw3* region in family D, *Dw1* in family C, and *SbFL9.1* in families D and E) (Table 4), suggesting that the exotic parents of these families carry native recessive alleles for early flowering or dwarfing. No significant biparental QTL were detected outside the three major regions defined by Thurber *et al.* (2013) on chromosomes 6, 7, and 9. However, one QTL for tropical plant height (HT-MX) was detected at 12.703 Mb on chromosome 1 in GWAS models ABC and ABCD.

**Table 4:** Comparison of QTL Positional Estimates in Mb

Locus-chr (Actual Position)		*Ma1*-chr6 (40.267–40.277)	*Dw2*-chr6 (uncloned)	*Dw3*-chr7 (58.557–58.565)	*Dw1*-chr9 (uncloned)	*SbFL9.1*-chr9 (uncloned)
Multiparental						
ABCDE	GWAS	42.073	42.073	**58.543**	57.109	58.554
ABCDE	Linkage	**40.203**	44.300	58.294	56.915	58.842
ABCD	GWAS	40.664	42.782	58.680	56.887	58.842
ABCD	Linkage	40.121	42.611	58.504	56.915	58.842
ABC	GWAS	40.038	42.782	58.680	57.493	58.638
ABC	Linkage	40.121	42.611	58.504	57.109	58.842
AB	GWAS	42.387	44.340	NS	57.109	58.679
AB	Linkage	40.047	44.300	NS	57.109	58.679
Biparental						
A	Linkage	42.487	44.445	NS	NS	NS
B	Linkage	40.052	44.340	NS	57.109	58.679
C	Linkage	40.038	NQ	58.680	NQ	58.554
D	Linkage	40.664	41.232	NQ	56.886	NQ
E	Linkage	**40.203**	42.648	58.228	57.599	NQ

QTL positions for *Ma1* and *SbFL9.1* are shown for the FL-IL trait, and QTL positions for *Dw1*, *Dw2*, and *Dw3* are shown for the HT-IL trait. Only the first effect in each region to enter the model is shown. The closest QTL to the cloned *Ma1* and *Dw3* loci are shown in bold. NS, not segregating; NQ, no QTL detected. All genomic positions refer to version 2.1 of the sorghum reference genome.

### QTL colocalization with cloned loci

#### Ma1:

**Figure 4:**
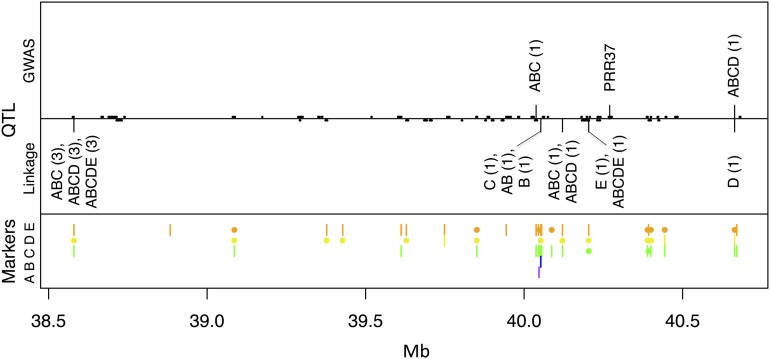
QTL for temperate flowering time (FL-IL) in the *Ma1* region of sorghum chromosome 6. Top and middle panels display GWAS and linkage QTL labeled with the families used in the model and, in parentheses, the step that the QTL entered the model. Predicted genes on the plus and minus strands are shown as black bars above and below the horizontal line. The PRR37 gene at 40.3 Mb underlies the *Ma1* locus. The lower panel shows SNP segregation across the five families. Vertical lines represent segregating SNPs and filled circles represent SNPs fixed for the alternate allele within a family.

The *Ma1* (*SbPRR37)* locus lies between 40.267 and 40.277 Mb on chromosome 6, and at least six *ma1* alleles confer photoperiod insensitivity through independent mutations in the *PRR37* gene (Murphy *et al.* 2011; R. Klein, personal communication). All FL-IL models include a *Ma1*-linked QTL (Table 4). Linkage models for FL-IL in all families (ABCDE) and family E alone both identify the closest SNP to *Ma1*, at 40.204 Mb (Figure 4). Because this SNP is only segregating in family E, it was not selected for any of the GWAS models. FL-IL QTL in four of the five biparental families fall within 0.4 Mb of PRR37. In general, the GWAS models for FL-IL fare relatively poorly. For example, the GWAS-ABCDE model identifies a SNP nearly 2 Mb away from *Ma1* at 42.073 Mb.

There are likely two inter-related problems affecting the GWAS FL-IL models. The first is that there are no SNPs in the dataset segregating in all five populations, even though each population apparently harbors a *Ma1* QTL. The second problem is that there appears to be an allelic series at *Ma1*. Additive effect estimates of the *Ma1*-linked FL-IL QTL in the *kafir* families A and B range from 5.3 to 7.1 d, whereas effect estimates for families C, D, and E range from 18.7 to 19.9 d (Table 5).

**Table 5:** QTL effect estimates in days *(Ma1* and *SbFL9.1)* and cm (*Dw3* and *Dw1*)

QTL	Model			Effects					
chr-Mb	Type	Families	Step	A	B	C	D	E	Combined
***Ma1:* chr6, 40.27 Mb**									
6-42.07	GWAS	ABCDE	1	0	0	1	1	1	18.3
6-40.66	GWAS	ABCD	1	0	0	1	2	1	19.2
6-40.04	GWAS	ABC	1	0	0	1	—	—	14.3
6-42.39	GWAS	AB	1	1	1	—	—	—	13.8
6-40.20	JL	ABCDE	1	5.0	−0.6	3.8	16.3	12.1	—
6-38.58	JL	ABCDE	3	NS	7.8	14.2	4.0	7.3	—
6-40.12	JL	ABCD	1	5.0	−17	3.4	17.0	—	—
6-38.58	JL	ABCD	3	NS	24.2	14.5	3.4	—	—
6-40.12	JL	ABC	1	5.0	−17	3.4	—	—	—
6-38.58	JL	ABC	3	NS	24.2	14.5	—	—	—
6-40.05	JL	AB	1	5.0	7.1	—	—	—	—
6-varies	Linkage	—	1	5.3	7.1	18.7	19.9	19.3	—
***Dw3:* chr7, 58.56 Mb**									
7-58.54	GWAS	ABCDE	3	0	0	1	2	1	45.3
7-58.68	GWAS	ABCD	3	0	0	1	0	—	40.7
7-58.68	GWAS	ABC	2	0	0	1	—	—	40.7
7-58.29	JL	ABCDE	3	NS	NS	37.6	2.3	43.1	—
7-58.50	JL	ABCD	3	NS	NS	38.3	3.1	—	—
7-58.50	JL	ABC	2	NS	NS	38.3	—	—	—
7-varies	Linkage	—	Varies	NS	NS	42.3	NQ	52.7	—
***Dw1:* chr9, ∼57 Mb**									
9-57.11	GWAS	ABCDE	1	0	1	2	1	1	30.2
9-56.89	GWAS	ABCD	1	0	0	0	1	—	77.1
9-57.49	GWAS	ABC	3	2	1	0	—	—	27.3
9-57.11	GWAS	AB	2	0	1	—	—	—	21.9
9-56.92	JL	ABCDE	2	NS	28.4	2.0	76.8	22.9	—
9-56.92	JL	ABCD	1	NS	28.4	3.5	77.6	—	—
9-57.11	JL	ABC	3	NS	28.7	3.8	—	—	—
9-57.11	JL	AB	2	NS	22.4	—	—	—	—
9-varies	Linkage	—	Varies	NS	21.9	NQ	76.5	28.9	—
***SbFL9.1:* chr9, ∼59 Mb**									
9-58.55	GWAS	ABCDE	2	0	1	1	2	1	6.5
9-58.84	GWAS	ABCD	2	0	1	1	0	—	6.2
9-58.64	GWAS	ABC	2	0	1	1	—	—	7.0
9-58.68	GWAS	AB	2	0	1	—	—	—	6.2
9-58.84	JL	ABCDE	2	NS	5.4	7.1	0.7	4.5	—
9-58.84	JL	ABCD	2	NS	5.4	7.1	0.6	—	—
9-58.84	JL	ABC	2	NS	5.4	7.1	—	—	—
9-58.68	JL	AB	2	NS	5.7	—	—	—	—
9-varies	Linkage	—	Varies	NS	5.6	8.7	NQ	NQ	—

The first SNP selected in the GWAS-ABCDE FL-IL model has an effect of 18.3 d, is segregating in families C, D, and E, and is fixed for the early allele in *kafir* families A and B. This is the closest SNP to *Ma1* that displays this pattern. The second *Ma1*-linked SNP selected (the third SNP selected overall in the GWAS-ABCDE FL-IL model) has an effect of 5.9 d, is segregating in families A and B, and is fixed for the early allele in families C, D, and E (Table S4). Again, this is the closest SNP to *Ma1* that displays the pattern, but it is more than 4 Mb away. In trying to fit an allelic series at *Ma1*, the mapping resolution of the GWAS model suffers.

It is surprising that *kafir* families A and B show a *Ma1*-linked flowering time QTL at all, because these families are segregating for two presumed knockout alleles at *Ma1*: *Sbprr37*-1, which has a premature stop codon upstream of both the pseudoreceiver and CCT domains, and *Sbprr37-2*, which contains a nonsynonymous substitution in the pseudoreceiver domain but has an intact CCT domain. Our results suggest that *Sbprr37-2* homozygotes are 10–14 d later than *Sbprr37-1* homozygotes in Illinois (a = 5.3–7.1 d). In contrast, wild-type *SbPRR37* homozygotes are more than 1 month later than *Sbprr37-1* homozygotes in Illinois (a = 18.7–19.9 d). To our knowledge, this constitutes the first direct evidence of a functional allelic series at *Ma1*, the major locus controlling photoperiod sensitivity in sorghum.

Additional flowering time QTL linked to *SbPRR37* may contribute to *Ma1*. Joint linkage models ABC, ABCD, and ABCDE place a QTL at 38.580 Mb, 2 Mb proximal to *SbPRR37*. The effects of this QTL are largest in families B and C, which also place their first FL-IL QTL proximal to *Ma1* in the biparental analyses (Table 4 and Table 5). Sorghum displays suppression of recombination for much of chromosome 6 (Wang *et al.* 2013), making it difficult to genetically separate the effects of *SbPRR37* from other potential contributors to *Ma1*.

#### Dw3:

**Figure 5:**
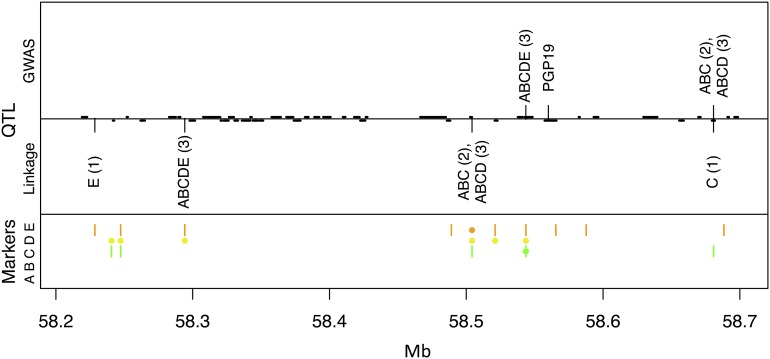
QTL for temperate plant height (HT-IL) in the *Dw3* region of sorghum chromosome 7. Information in the top, middle, and bottom panels is the same as in Figure 4. The PGP19 gene at 58.557 Mb underlies the *Dw3* locus.

The sorghum *Dw3* locus is encoded by *SbPGP19*, an auxin efflux carrier lying between 58.557 and 58.565 Mb on chromosome 7; the unstable recessive *dw3* allele confers dwarfism due to a tandem duplication in the fifth exon at 58.558 Mb (Multani *et al.* 2003). GWAS-ABCDE plant height models for both HT-IL and HT-MX identify the same SNP at 58.544 Mb (Figure 5, Table S4), which is less than 15 kb from the causal mutation and is segregating in the two families (C and E) that contain significant *Dw3*-linked plant height QTL in the biparental analyses (Table 4). Two SNPs in our dataset are closer to the causal mutation, but both of these are segregating in just one family (Table S1). Both the joint linkage models and the biparental linkage models in families C and E place the *Dw3* QTL much farther away from the causal mutation (Figure 5).

The SNP at 58.544 Mb selected for GWAS height models is fixed for the “tall” allele in families A and B, and is fixed for the short allele in family D (Table 4). Therefore, this SNP begins to explain some of the variance that is fixed between families. This situation never occurs in reference design populations like the NAM population, in which only fixation of the reference allele can occur within families. Moreover, the SNP at 58.544 Mb captures the underlying biological reality better than a marker for the tandem duplication itself would do. Family D is segregating for the tandem duplication but not the QTL, because it carries an independent loss-of-function mutation in *Dw3* (Barrero Farfan *et al.* 2012).

### QTL mapping of uncloned loci

#### Dw1:

**Figure 6:**
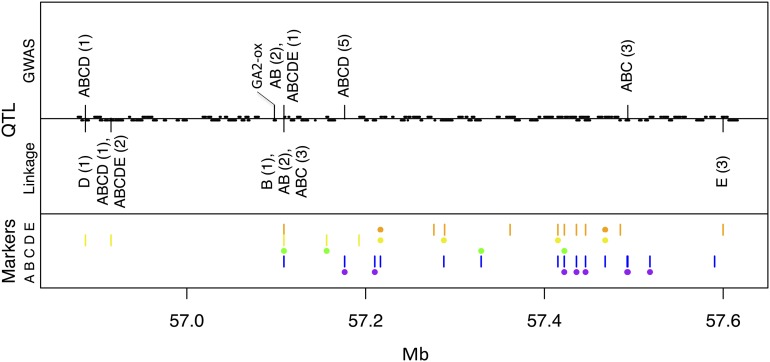
QTL for temperate plant height (HT-IL) in the *Dw1* region of sorghum chromosome 9. Information in the top, middle, and bottom panels is the same as in Figure 4. The GA2-ox candidate gene at 57.098 Mb is shown.

The uncloned *Dw1* locus maps to ∼57 Mb on chromosome 9 (Morris *et al.* 2012; Thurber *et al.* 2013). Several investigators have suggested that a nearby GA-2-oxidase at 57.097–57.099 Mb might underlie the *Dw1* locus (Brown *et al.* 2008; Morris *et al.* 2012). Several GWAS models (AB, ABCDE) and linkage models (B, AB, ABC) for HT-IL identify the same SNP at 57.109 Mb, which is the closest SNP in our dataset to the GA2-oxidase. This SNP is segregating in families B, D, and E and is fixed for the tall and short alleles in families A and C, respectively (Figure 6). Most models for HT-MX map *Dw1* slightly distal, at 57.176 Mb (Figure S3). GA-2-oxidases are responsible for the catabolism of active gibberellic acid, and gain-of-function mutations lead to dominant dwarfism (Busov *et al.* 2003).

#### SbFL9.1:

**Figure 7:**
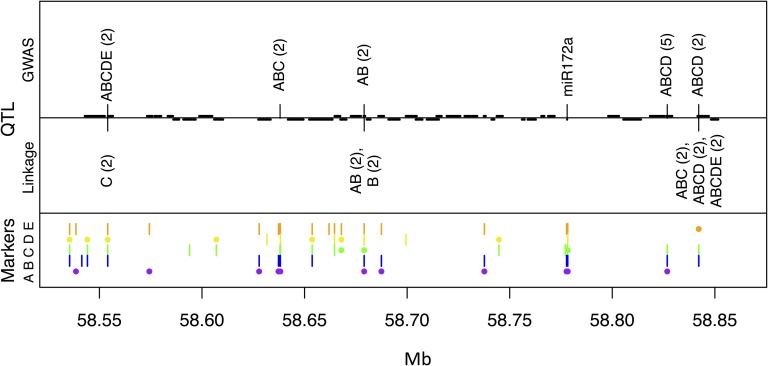
QTL for temperate flowering time (FL-IL) in the *SbFL9.1* region of sorghum chromosome 9, distal to *Dw1*. Information in the top, middle, and bottom panels is the same as in Figure 4. The position of the miR172a candidate gene at 58.778 Mb is shown.

A recent study (Thurber *et al.* 2013) reported a flowering QTL several Mb distal to *Dw1*, which we refer to here as *SbFL9.1*. Linkage disequilibrium between the most significant SNPs for *Dw1* and *SbFL9.1* is low, suggesting that they represent separate QTL rather than a single QTL with pleiotropic effects on plant height and flowering time (Thurber *et al.* 2013). All joint linkage models for FL-IL that contain families B and C, in which the QTL appears to be segregating, identify the same SNP at 58.842 Mb (Figure 7). One candidate for this QTL is a *miR172a* transcript at 58.778 Mb; this microRNA family targets AP2 transcription factors that play a role in plant flowering induction (Lauter *et al.* 2005).

#### Dw2:

Early genetic work in sorghum established that *Ma1* was linked to the dwarfing locus *Dw2* (Quinby 1974) and numerous independent studies have estimated the position of *Dw2* at several Mb distal to *Ma1*, at ∼43–45 Mb on chromosome 6 (Klein *et al.* 2008; Morris *et al.* 2012; Thurber *et al.* 2013). Results from the present study suggest that the most plausible location for *Dw2* is the interval between 44.30 and 44.45 Mb, which contains a HT-IL GWAS QTL for model AB and linkage QTL for models A, B, AB, and ABCDE (Table 4; Figure S4). The ABCDE-GWAS models for both FL-IL and HT-IL fit the same SNP at 42.073 Mb, far from any QTL detected in the biparental models. These and other QTL for temperate plant height (HT-IL) between 41 and 43 Mb may be synthetic associations resulting from the fusion of *Ma1* and *Dw2*, because they occur approximately halfway between *Ma1* and the presumed location of *Dw2* are not replicated in the tropical environment (Figure S4 and Figure S5) and only occur with the inclusion of photoperiod-sensitive families CDE.

#### Effects of genetic architecture and model choice on mapping performance:

We used the cloned loci *Dw3* and *Ma1* to assess the performance of two classes of multiparental models (joint linkage and GWAS) and four subsets of families with decreasing interfamily and intrafamily relatedness (AB, ABC, ABCD, and ABCDE). The GWAS model performs best at the *Dw3* locus, whereas the joint linkage model performs best at the *Ma1* locus, and in both cases the ABCDE model that includes all families performs best. Both *Ma1* and *Dw3* harbor genetic heterogeneity in the form of multiple loss-of-function mutations. However, the two loss-of-function mutations at *Dw3* appear to be functionally equivalent, because family D is segregating for both of them and does not have a *Dw3* QTL. In contrast, the two loss-of-function mutations at *Ma1* appear functionally distinct, with families A and B segregating for a small-effect QTL and families C, D, and E segregating for a large-effect QTL. The GWAS model cannot fit the *Ma1* allelic series in a single step but fits the multiple knockout mutations at *Dw3* with a synthetic association that captures the underlying biological reality better than either causal mutation.

This study contains several orders of magnitude fewer SNPs than previous maize studies that performed GWAS/joint linkage in the NAM population. The smaller number of SNPs reported here reflects a number of important limitations, including: the reduced nucleotide diversity of sorghum compared with maize; the reduced number of families compared with NAM (5 *vs.* 25); the use of partially isogenic families rather than a reference design; and the genotyping method used (GBS *vs.* whole genome resequencing). These are likely to be common limitations for other researchers working in breeding programs of self-pollinated crops.

Both GWAS and joint linkage models were prone to inaccurate mapping when presented with genetic heterogeneity. The successful tagging of *Dw3* by the GWAS model is probably fortuitous because our cost-effective, low-coverage GBS genotyping yielded far too few SNPs to capture all haplotypes segregating in these five families. Success of the GWAS model is less likely for high-frequency QTL like *Ma1*, especially in situations of marker limitation, because most SNPs are rare. Colocalization of GWAS and joint linkage QTL provide increased confidence in QTL position, because these models incorporate linkage disequilibrium information at different levels.

## Supplementary Material

Supporting Information
