# Supplementary material for: Multiparental Mapping of Plant Height and Flowering Time QTL in Partially Isogenic Sorghum Families
Source: G3 (Bethesda). 2014 Sep 1;4(9):1593–602. doi: 10.1534/g3.114.013318 (PMC4169151; doi:10.1534/g3.114.013318)
Supplement: Supporting Information [file supp_4_9_1593__index.html]

Supporting Information 

# Multiparental Mapping of Plant Height and Flowering Time QTL in Partially Isogenic Sorghum Families

## Supporting Information for Higgins *et al.*, 2014

**Files in this Data Supplement:**

- Supporting Information - Figures S1-S4 and Tables S1-S5 (PDF, 483 KB)
- Figure S1 - Raw and calculated values for days to anthesis in the temperate environment (FL‐IL) (PDF, 404 KB)
- Figure S2 - Temperate flowering time (FL‐IL) distributions of lines with and without genotype data in each family (PDF, 392 KB)
- Figure S3 - QTL for tropical plant height (HT‐MX) in the *Dw1* region of sorghum chromosome 9 (PDF, 141 KB)
- Figure S4 - QTL for temperate plant height (HT‐IL) in the *Dw2* region of sorghum chromosome 6 (PDF, 164 KB)
- Figure S5 - QTL for tropical plant height (HT‐MX) in the *Dw2* region of sorghum chromosome 6 (PDF, 135 KB)
- Table S1 - Segregation of 9139 SNPs across families A‐E (.xlsx, 305 KB)
- Table S2 - Raw and modeled phenotypes (.xlsx, 88 KB)
- Table S3 - Phenotypic variance components (.xlsx, 9 KB)
- Table S4 - QTL models (.xlsx, 13 KB)
- Table S5 - Raw genotypes (.zip, 8.8 MB)
