# Supplementary material for: Multiparental Mapping of Plant Height and Flowering Time QTL in Partially Isogenic Sorghum Families
Source: G3 (Bethesda). 2014 Sep 1;4(9):1593–602. doi: 10.1534/g3.114.013318 (PMC4169151; doi:10.1534/g3.114.013318)
Supplement: Supporting Information [file supp_4.9.1593_013318SI.pdf]

G3 · Genes | Genomes | Genetics

Multi-parental mapping of plant height and flowering time QTL in partially isogenic sorghum families

Higgins RH*§1, Thurber CS§, Assaranurak I*2, and Brown PJ*§

*Department of Crop Sciences, University of Illinois, Urbana, IL, USA 61801
§Energy Biosciences Institute, University of Illinois, Urbana, IL, USA 61801

[1] Current address: Department of Agronomy, Iowa State University, Ames, IA 50011
[2] Current address: Department of Plant Breeding and Genetics, Cornell University, Ithaca, NY 14850

Corresponding author: Patrick J Brown, 1408 IGB, 1206 W Gregory Drive, Urbana IL, 61801; (217)-333-8182; pjb34@illinois.edu

DOI: 10.1534/g3.114.013318

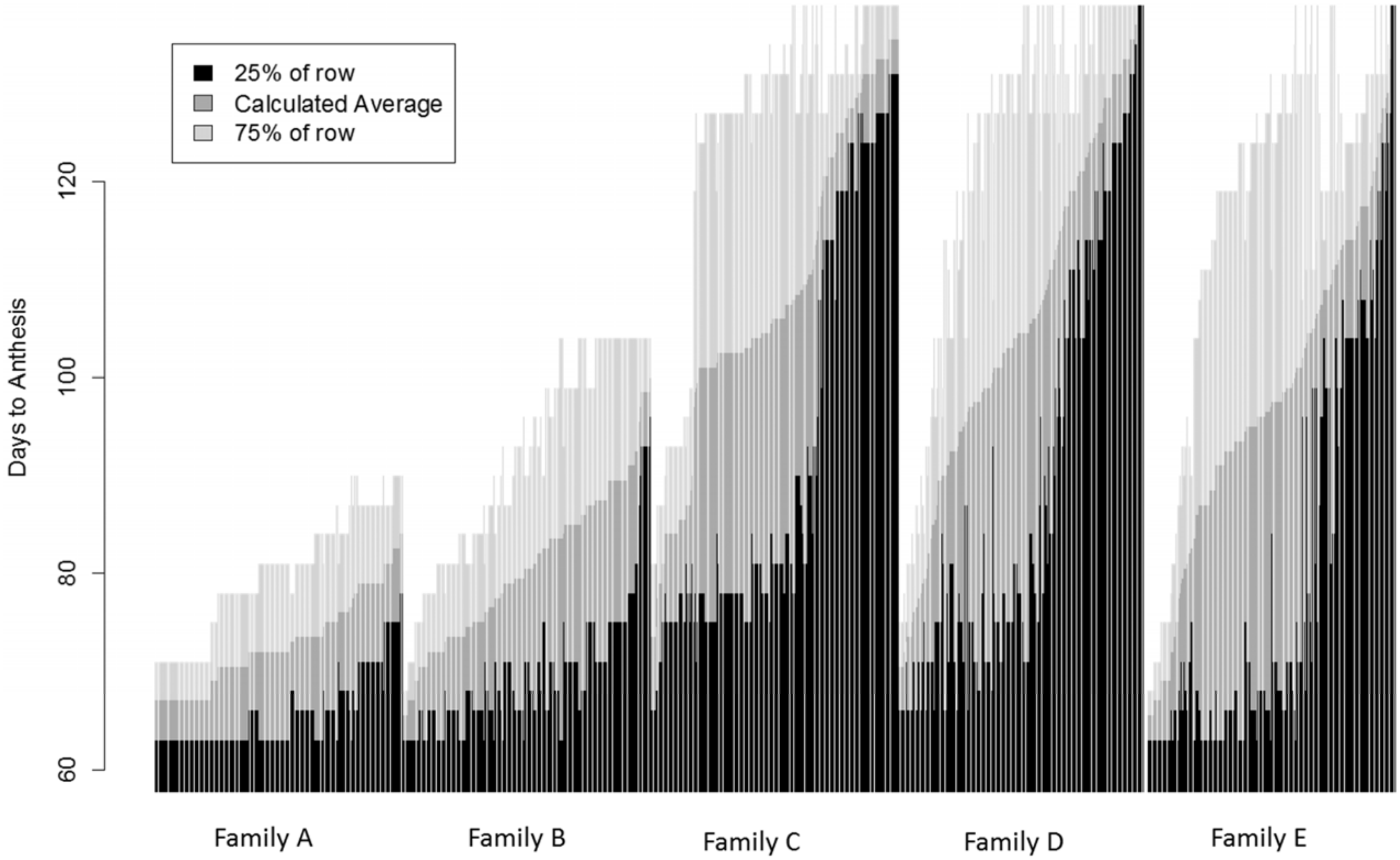

Figure S1 Raw and calculated values for days to anthesis in the temperate environment (FL-IL).

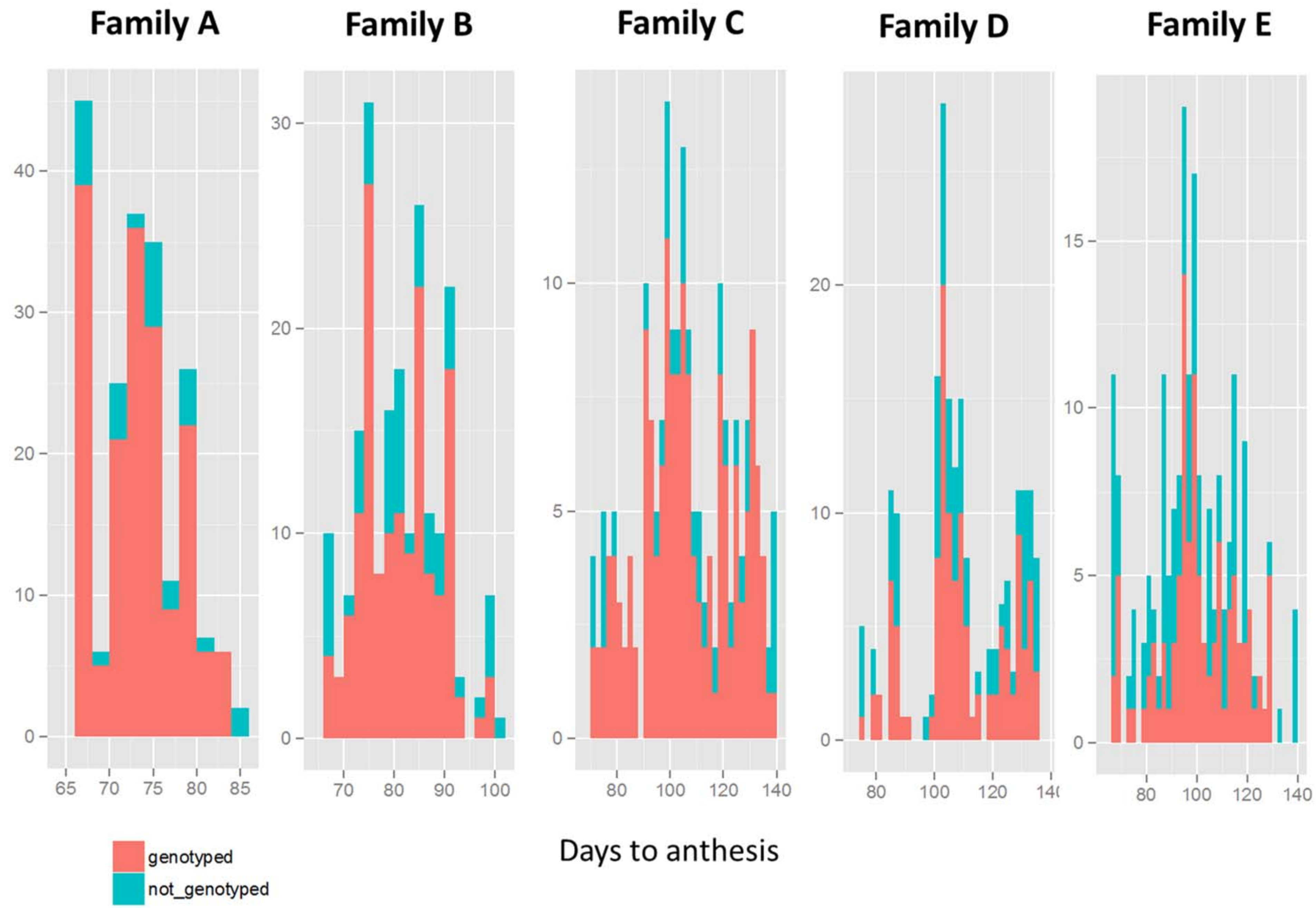

Figure S2 Temperate flowering time (FL-IL) distributions of lines with and without genotype data in each family.

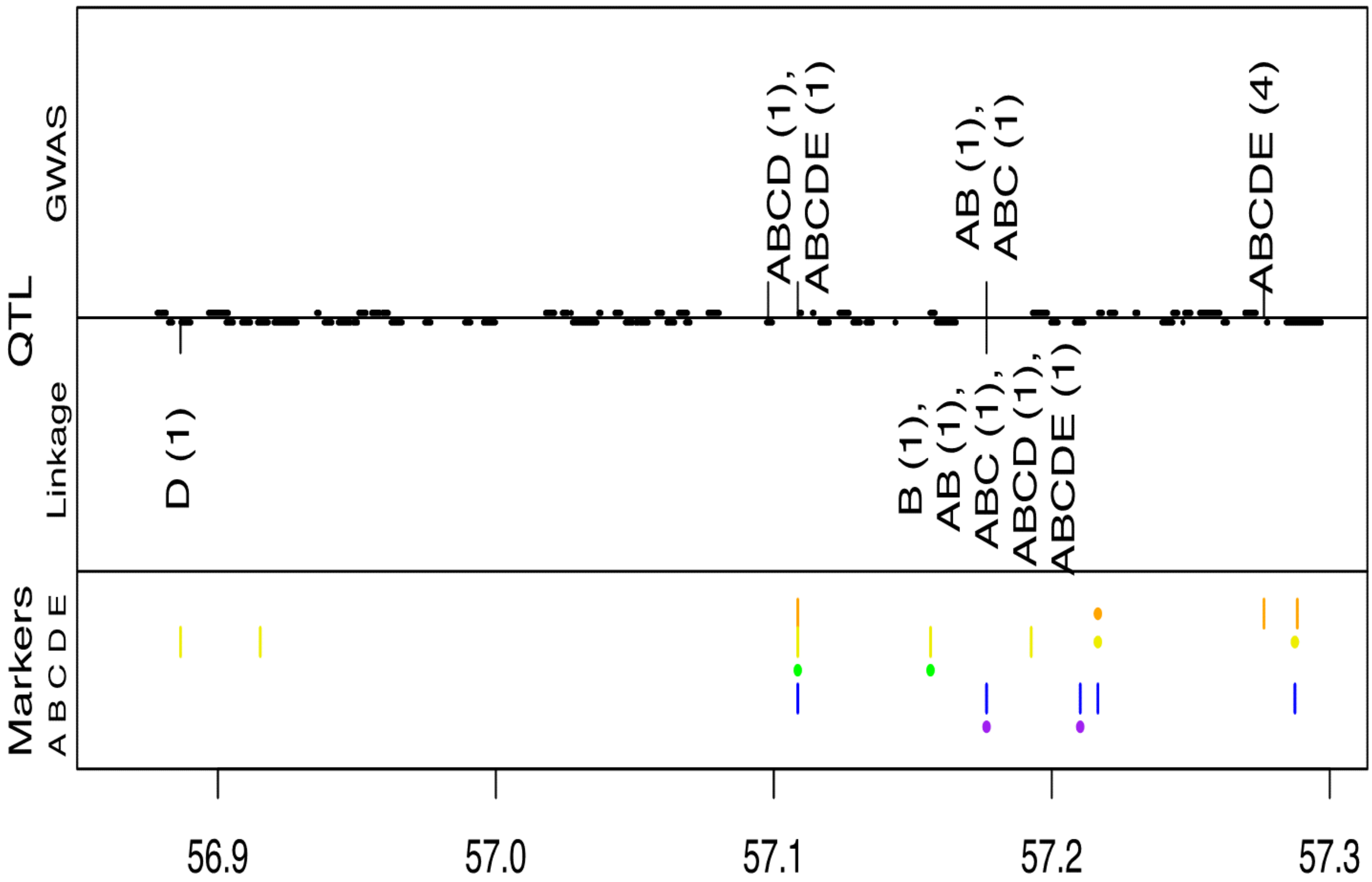

Figure S3 QTL for tropical plant height (HT-MX) in the *Dw1* region of sorghum chromosome 9. Information in the top, middle, and bottom panels is the same as in Figure 4.

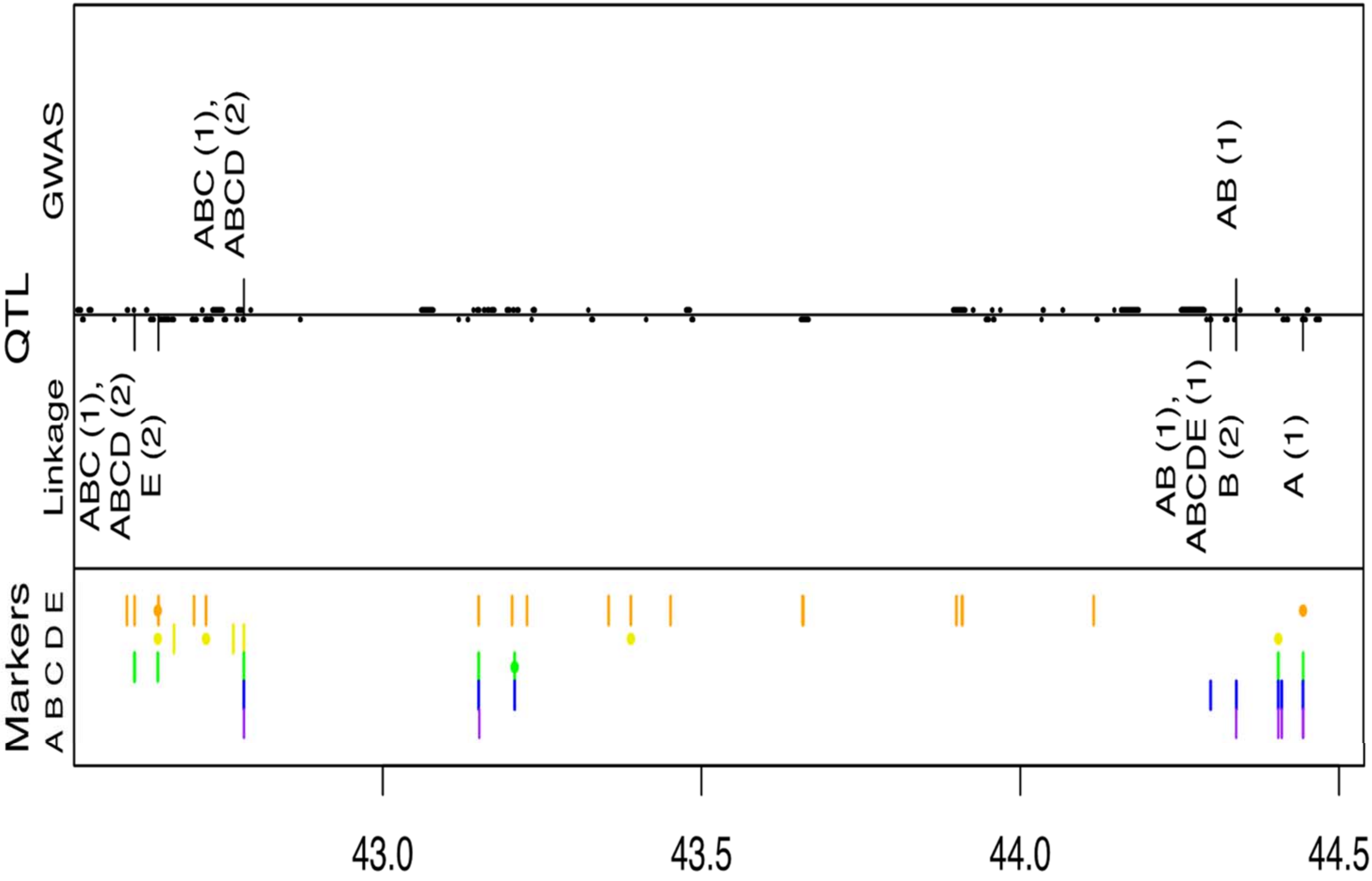

Figure S4 QTL for temperate plant height (HT-IL) in the *Dw2* region of sorghum chromosome 6. Information in the top, middle, and bottom panels is the same as in Figure 4.

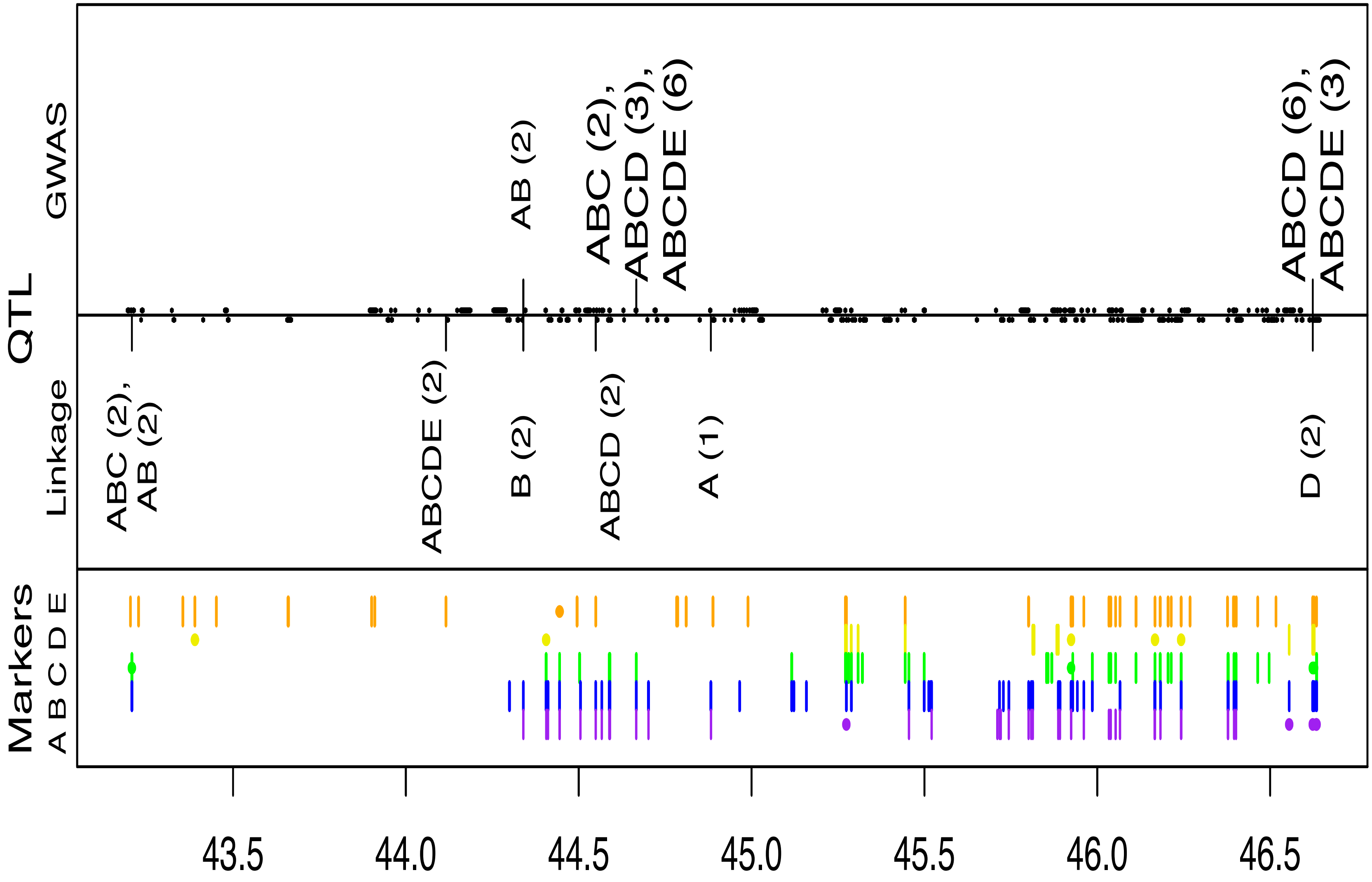

Figure S5 QTL for tropical plant height (HT-MX) in the *Dw2* region of sorghum chromosome 6. Information in the top, middle, and bottom panels is the same as in Figure 4.

Tables S1-S5

Available for download as Excel files at http://www.g3journal.org/lookup/suppl/doi:10.1534/g3.114.013318/-/DC1

Table S1 Segregation of 9139 SNPs across families A-E. Marker segregation by family: 0 = fixed for reference allele, 1 = segregating; 2 = fixed for alternate allele.

Table S2 Raw and modeled phenotypes. Raw phenotypic data ("_raw") and phenotypic values used for QTL analysis ("_out"). For FL-IL, days at which each row reached 25% and 75% anthesis are also shown.

Table S3 Phenotypic variance components. REML estimates of variance components for each phenotype, obtained using the lmer() function in R

Table S4 QTL models. Markers and p-values for each step in each QTL model.

Table S5 Raw genotypes. Genotypes used for QTL analysis. 0= fixed for reference allele; 1=heterozygous; 2= fixed for alternate allele; NA = missing.
