# Supplementary figures and images for: Multiparental Mapping of Plant Height and Flowering Time QTL in Partially Isogenic Sorghum Families
Source: G3 (Bethesda). 2014 Sep 1;4(9):1593–602. doi: 10.1534/g3.114.013318 (PMC4169151; doi:10.1534/g3.114.013318)

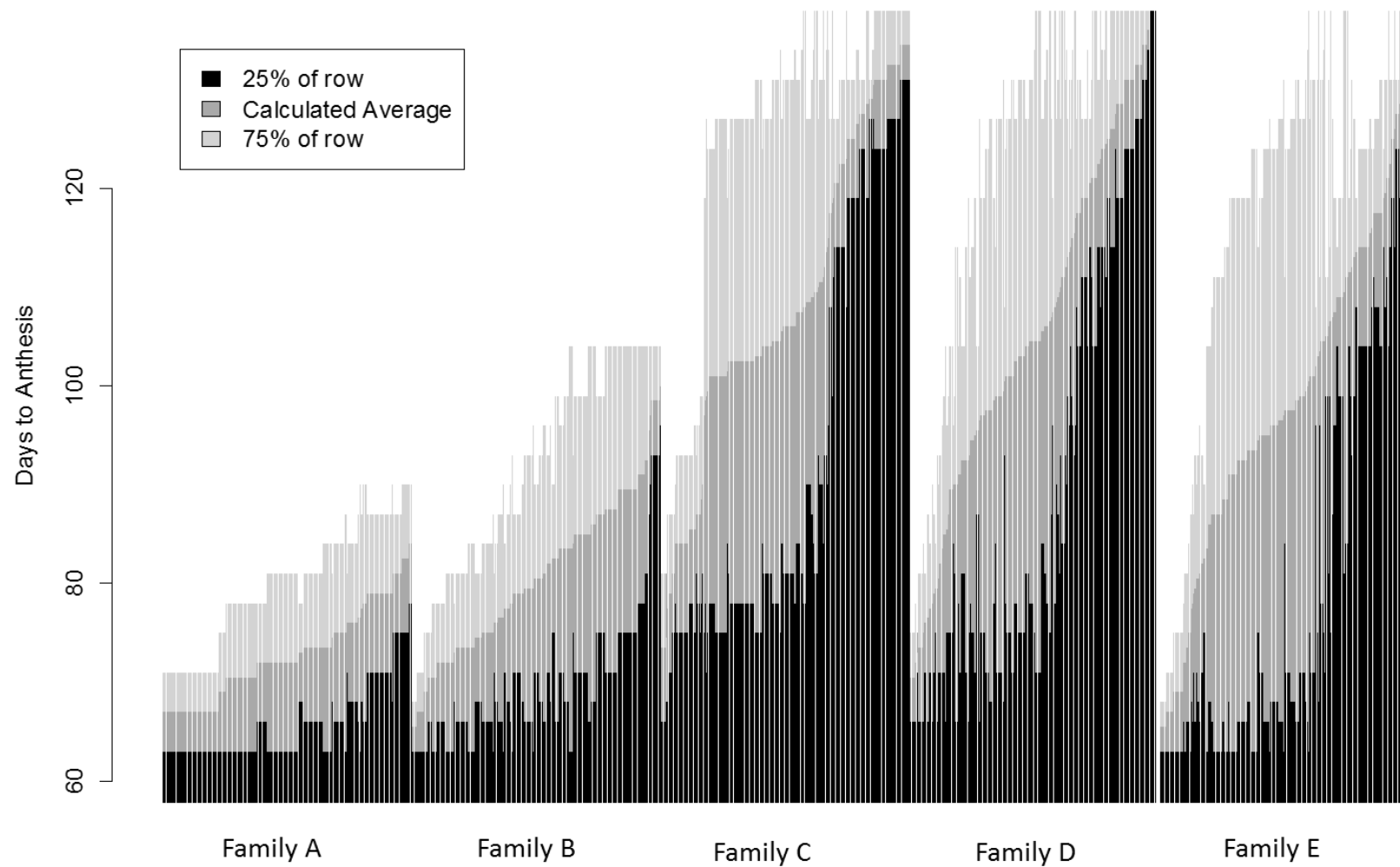

**Figure S1** Raw and calculated values for days to anthesis in the temperate environment (FL-IL).

Supplement: Supporting Information [file supp_4.9.1593_FigureS1.pdf]
