# Supplementary material for: Multiparental Mapping of Plant Height and Flowering Time QTL in Partially Isogenic Sorghum Families
Source: G3 (Bethesda). 2014 Sep 1;4(9):1593–602. doi: 10.1534/g3.114.013318 (PMC4169151; doi:10.1534/g3.114.013318)
Supplement: Supporting Information [file supp_4.9.1593_FigureS2.pdf]

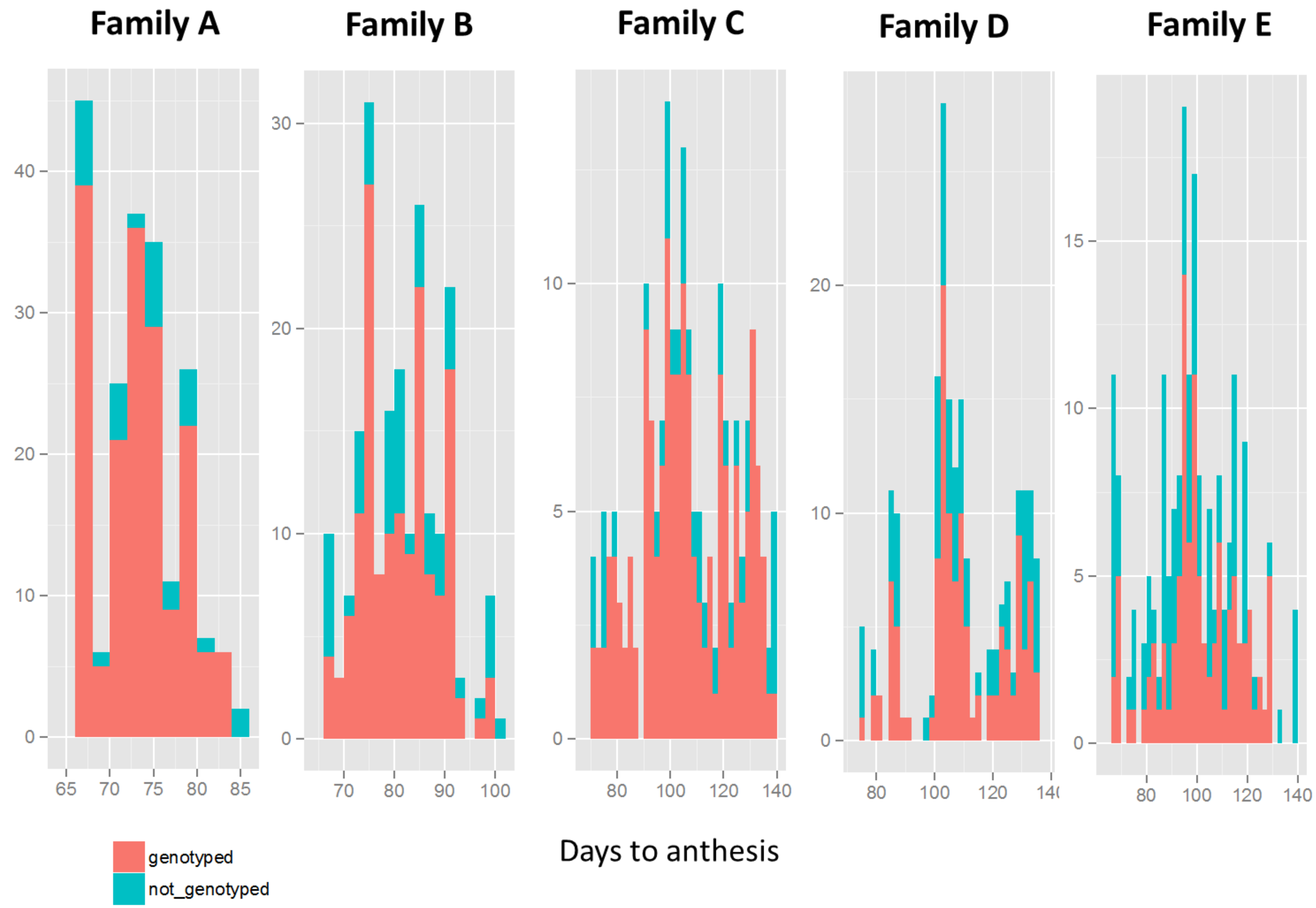

Figure S2 Temperate flowering time (FL-IL) distributions of lines with and without genotype data in each family.
