# Supplementary material for: Multiparental Mapping of Plant Height and Flowering Time QTL in Partially Isogenic Sorghum Families
Source: G3 (Bethesda). 2014 Sep 1;4(9):1593–602. doi: 10.1534/g3.114.013318 (PMC4169151; doi:10.1534/g3.114.013318)
Supplement: Supporting Information [file supp_4.9.1593_FigureS3.pdf]

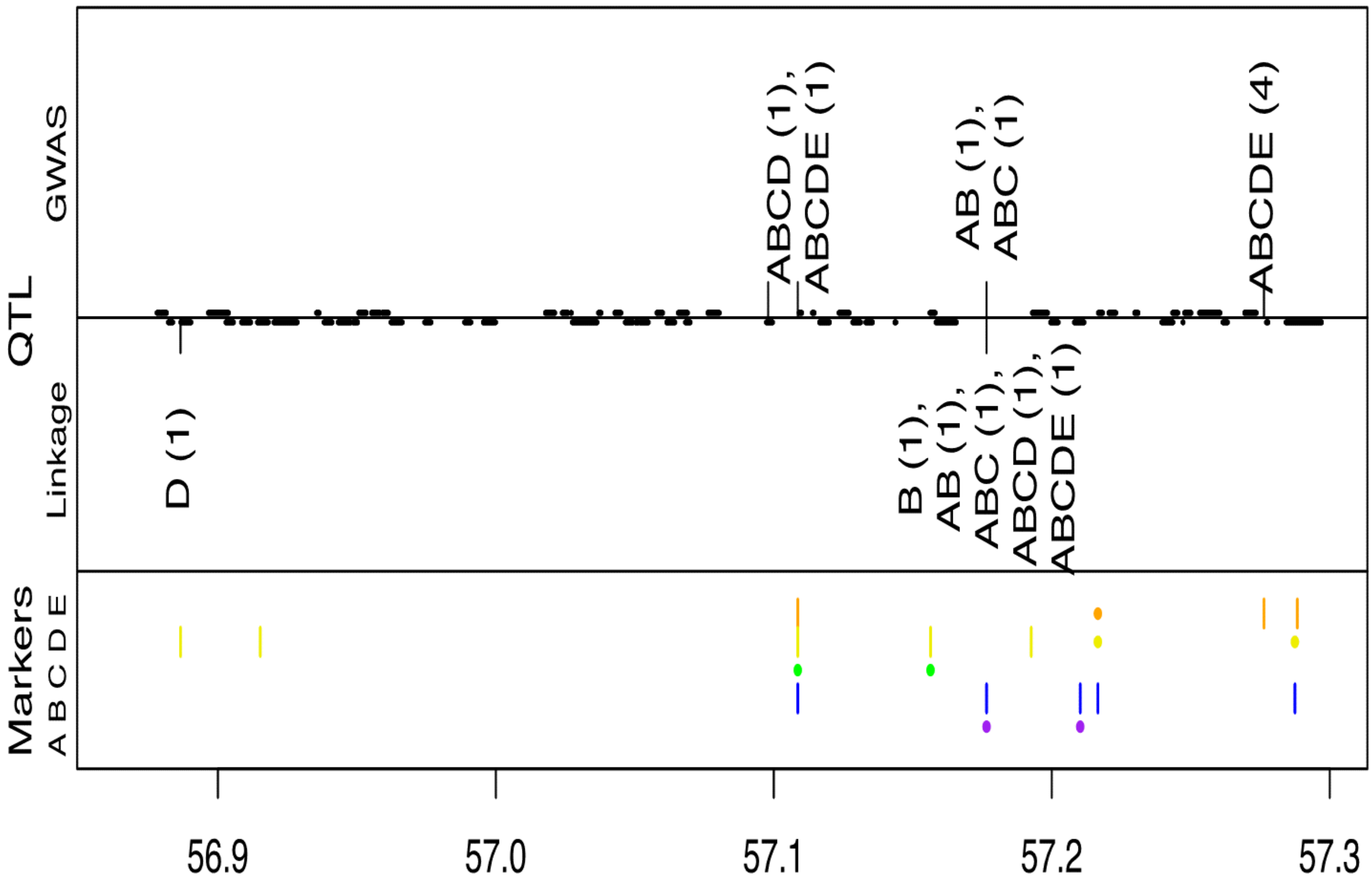

Figure S3 QTL for tropical plant height (HT-MX) in the *Dw1* region of sorghum chromosome 9. Information in the top, middle, and bottom panels is the same as in Figure 4.
